# Long noncoding RNA LINC00261 upregulates ITIH5 to impair tumorigenic ability of pancreatic cancer stem cells

**DOI:** 10.1038/s41420-021-00575-0

**Published:** 2021-08-26

**Authors:** Lijuan Zou, Hengpeng He, Zhiguo Li, Ou Chen, Xiukun Jia, Hao Zhang

**Affiliations:** Clinical Laboratory, The Fourth Affiliated Hospital of China Medical University, Shenyang, 110000 P. R. China

**Keywords:** Cancer, Diseases

## Abstract

Long noncoding RNAs (lncRNAs) are implicated tumor development in a range of different cancers, including pancreatic cancer (PC). Cancer stem cells (CSCs), a drug-resistant cancer cell subset, drive tumor progression in PC. In this work, we aimed to investigate the mechanism by which lncRNA LINC00261 affects the biological functions of CSCs during the progression of PC. Microarray analysis of differentially expressed genes and lncRNAs suggested that LINC00261 is downregulated in PC. Both LINC00261 and ITIH5 were confirmed to be downregulated in PC cells and PC stem cells. Gain-of-function and loss-of-function investigations were performed to analyze their effects on cell proliferation, drug resistance, cell cycle distribution, self-renewal, invasion, and ultimately overall tumorigenicity. These experiments revealed that the expression of stem cell markers was reduced, and cell proliferation, self-renewal ability, cell invasion, drug resistance, and tumorigenicity were all suppressed by upregulation of LINC00261 or ITIH5. The results of dual-luciferase reporter gene, ChIP, and RIP assays indicated that LINC00261 binds directly to GATA6, increasing its activity at the ITIH5 promoter. The presence of LINC00261 and GATA6 inhibited the self-renewal and tumorigenesis of PC stem cells, while silence of ITIH5 rescued those functions. Collectively, this study identifies the tumor suppressive activity of LINC00261 in PC, showing that this lncRNA limits the functions of PC stem through an ITIH5/GATA6 regulatory pathway.

## Introduction

Pancreatic cancer (PC) is an aggressive malignancy with high morbidity and mortality due to the fact that few effective therapies are available [1, 2]. Despite recent advances in surgery, immunotherapy, chemotherapy, and radiotherapy, the prognosis of PC patients often remains poor due to a lack of effective diagnostic biomarkers [3–5]. Cancer stem cells (CSCs) are drug-resistant cancer cell subsets responsible for tumor progression, metastasis, and relapse [6, 7]. CSCs, characterized by multipotency, have a high self-renewal capacity and carcinogenicity, playing a key role in the clinical response to immunotherapy [8]. The chemotherapy gemcitabine is regarded as a relatively effective treatment for advanced PC [9]. However, due to the invasive and metastatic characteristic, when coupled with a high level of genomic instability [10], further research is required to identify novel therapeutic targets to prevent tumor development and metastasis in PC [11]. Several studies have indicated the important involvement of a series of long noncoding RNAs (lncRNAs) in the progression and treatment of PC [12–14]. For example, aberrant expression of lncRNA HOTTIP enhanced tumor proliferation and invasion, as well as gemcitabine resistance, by modulating the transcription factor homeobox A13 (HOXA13) in PC [15].

LncRNAs, are important modulators in cancers, playing roles in both oncogenic and tumor suppressive pathways [16]. Strikingly, lncRNAs interact with transcription factors in core regulatory networks to participate in the cancer development [17–19]. The zinc-finger transcription factor, GATA binding protein 6 (GATA6), displays a key role in enhancing or suppressing tumor progression, with the role played depending on the tumor origins [20–22]. In addition, another study has reported that GATA6 enhanced the progression of PC by activating the Wnt signaling pathway via regulating DKK1 [23]. Inter-α-trypsin inhibitors (ITIs) are a family of serine protease inhibitors which have one light chain and various heavy chains [24, 25]. ITI heavy chain 5 (ITIH5) was revealed to have close correlation with tumor suppression in diverse kinds of cancers [26–28]. LINC00261 is reported to act as a tumor suppressive lncRNA in various cancers including gastric cancer and non-small cell lung cancer [29, 30]. Although LINC00261 have been proposed to be a novel prognostic marker for PC [31], its role and regulatory mechanism in PC remains elusive. Therefore, this study aims to investigate the possible functions of LINC00261, GATA6, and ITIH6 in PC, with the aim of identifying the molecular mechanisms by which each function in PC.

## Results

### LINC00261 and ITIH5 expressions are low in PC cells and PC CSCs

**Fig. 1:**
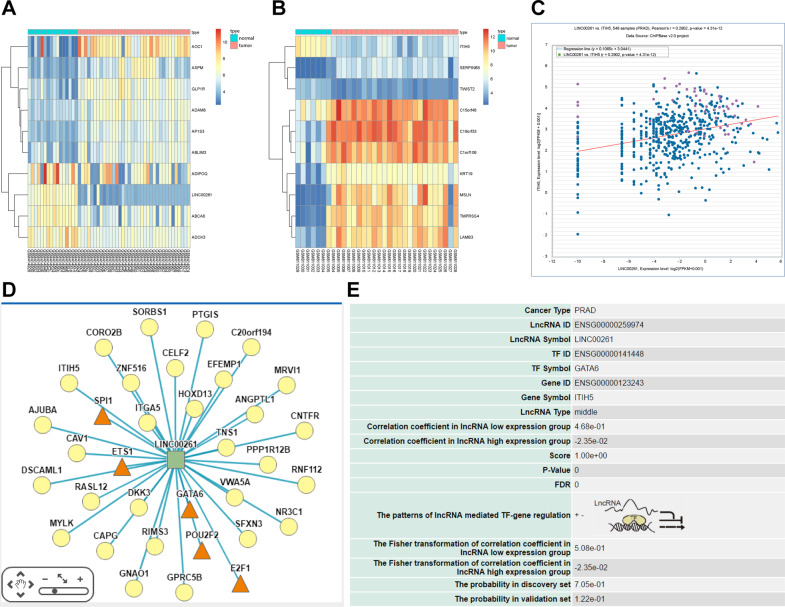
LINC00261 and ITIH5 are downregulated in PC. **A** The heatmap of GSE16515 expression dataset. **B** The heatmap of GSE32676 expression dataset. **C** Coexpression of LINC00261 and ITIH5 analyzed by ChIPBase website. **D** Possible regulatory mechanism of LINC00261 predicted by the lncMAP website. **E** Possible regulatory mechanism of LINC00261 regulating ITIH5 predicted by the lncMAP website. PC pancreatic cancer, ITIH5 inter-alpha-trypsin inhibitor heavy chain H5.

In order to examine LINC00261 and ITIH5 expression in PC, we conducted analysis of GSE16515 and GSE32676 datasets, revealing that the expression of LINC00261 and ITIH5 was downregulated in PC tissues relative to healthy control samples (Fig. 1A, B). According to the ChIPBase website (http://rna.sysu.edu.cn/chipbase/index.php), LINC00261 was positively correlated with ITIH5 in PC (Fig. 1C). The lncMAP website (http://bio-bigdata.hrbmu.edu.cn/LncMAP/search.jsp) was used to predict the possible regulatory mechanism of LINC00261 (Fig. 1D), with the results showing that LINC00261 may regulate the expression of ITIH5 through the transcription factor GATA6 in PC (Fig. 1E). These results indicate that LINC00261 is downregulated in PC tissues, while ITIH5 expression followed a similar pattern.

### PANC-1 stem cells exhibit decreased expression of LINC00261

**Fig. 2:**
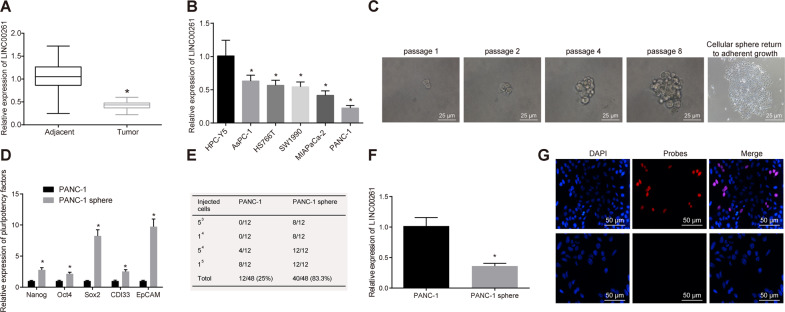
LINC00261 is downregulated in verified PANC-1 stem cells. **A** The expression of LINC00261 in PC tissues (*n* = 88) and adjacent normal tissues (*n* = 88) determined by RT-qPCR. *p < 0.05 vs. the adjacent normal tissues. **B** The LINC00261 expression in PANC-1, AsPC-1, HS 766T, SW1990, MIA PaCa-2, and HPC-Y5 cell lines examined by RT-qPCR. *p < 0.05 vs. the HPC-Y5 cell line. **C** The enriched cell sphere in PANC-1 cells by suspension culture and the adherent cells after normal culture observed under the microscope (400×, scale bar = 25 µm). **D** Expression of surface molecular markers Nanog, Oct4, Sox2, CDl33, and EpCAM in PANC-1 cell sphere examined by RT-qPCR. *p < 0.05 vs. the PANC-1 cells. **E** The tumorigenicity of PANC-1 cells and PANC-1 stem cells in nude mice, *n* = 5. *p < 0.05 vs. the PANC-1 cells. **F** LINC00261 expression in PANC-1 cells and PANC-1 stem cells examined by RT-qPCR. *p < 0.05 vs. the PANC-1 cell. **G** Cellular location of LINC00261 in PANC-1 cells examined by RNA-FISH (200×, scale bar = 50 µm). Each experiment was repeated three times and measurement data were expressed as mean ± standard deviation. Comparisons between two groups were analyzed by paired or independent sample *t* test and comparisons among multiple groups were analyzed by one-way ANOVA, followed by Tukey’s post hoc test. RNA-FISH RNA-fluorescence in situ hybridization.

Next, for exploring these findings experimentally, microarray data analysis was conducted, followed by reverse transcription quantitative polymerase chain reaction (RT-qPCR) for examining the expression of LINC00261 in PC tissues and adjacent normal tissues. The results (Fig. 2A) revealed that LINC00261 was significantly decreased in PC tissues relative to adjacent normal tissues (*p* < 0.05). Moreover, the expression of LINC00261 in five commonly used PC cell lines including PANC-1, AsPC-1, HS 766T, SW1990, MIA PaCa-2, and normal pancreatic epithelial cell line (HPC-Y5) was examined by RT-qPCR, revealing that the expression of LINC00261 is lower in all five PC cell lines compared with the HPC-Y5 cells (*p* < 0.05), with PANC-1 displaying the lowest expression (Fig. 2B). Hence, PANC-1 was selected for all further in vitro experiments.

Cell spheres in PANC-1 cells were enriched in suspension culture of a 96-well plate at a density of 1 cell/well by adopting a dilution method (Fig. 2C). To verify that the cell spheres were the expected stem cells, the expression of cell sphere stemness-related transcriptional factor (Nanog, Oct4, Sox2) and related surface molecular markers (CDl33 and EpCAM) in PANC-1 cells was determined by RT-qPCR. This revealed that, compared with PANC-1 cells, the PANC-1 spheres showed increased expression of the aforementioned surface molecular markers (*p* < 0.05, Fig. 2D), indicating the PANC-1 stem cells had been successfully enriched. Encouragingly, the cells of each generation were analyzed by flow cytometry, which detected 92.56% CD133-positive and 93.42% EpCAM-positive cells.

The tumorigenicity of the stem cells was determined in nude mice; PANC-1 cells and PANC-1 stem cells were continuously diluted and then injected in nude mice for tumor formation observation. The results from this assay suggested that the tumor formation ability of PANC-1 stem cells in nude mice was enhanced vs. PANC-1 cells (*p* < 0.05, Fig. 2E). This result revealed that the PANC-1 stem cells had been successfully isolated. The expression of LINC00261 in PANC-1 cells and PANC-1 stem cells was then determined by RT-qPCR. The results exhibited that compared with the PANC-1 cells, the LINC00261 expression in the PANC-1 stem cells decreased (*p* < 0.05, Fig. 2F). The cellular localization of LINC00261 in PANC-1 cells was analyzed by RNA-fluorescence in situ hybridization (FISH), and the results suggested LINC00261 was mostly located in the nucleus in PANC-1 cells (Fig. 2G). Taken together, these findings suggest that PANC-1 stem cells have a lower expression of LINC00261.

### LINC00261 inhibits the proliferation, self-renewal, invasion, and tumorigenicity of PC stem cells while also enhancing the sensitivity to gemcitabine

The above experiments suggest that LINC00261 is poorly expressed in PC stem cells, thus a gain-of-function experiment was designed to investigate the role of LINC00261 in the biological functions of PC stem cells.

**Fig. 3:**
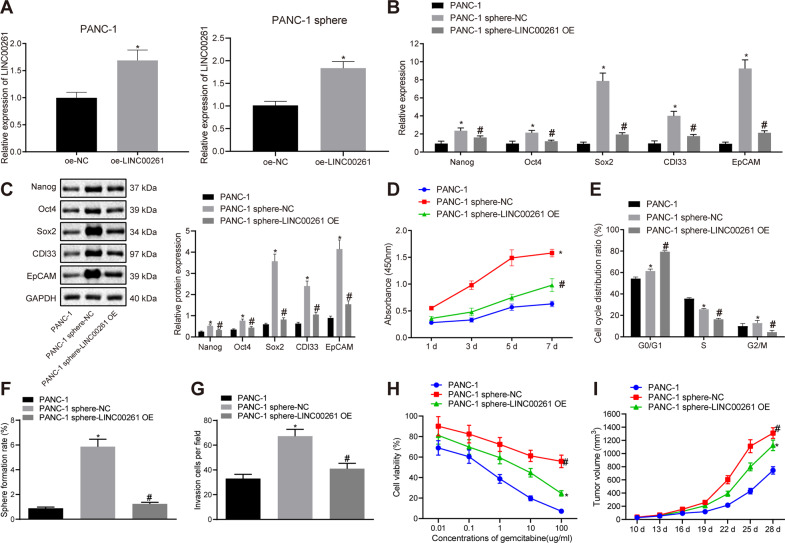
Upregulation of LINC00261 suppresses the proliferation, self-renewal, invasion and tumorigenicity while enhances the sensitivity for gemcitabine in PANC-1 cells and stem cells. **A** Transfection efficiency of LINC00261 overexpression in PANC-1 cells and stem cells determined by RT-qPCR. **B** Expression of stem cell markers in PANC-1 cells, the oe-NC group, and the oe-LINC00261 group determined by RT-qPCR. **C** The protein levels of stem cell markers in PANC-1 cell and stem cells measured using western blot analysis. **D** Cell proliferation of PANC-1 stem cell in PANC-1 cells, the oe-NC group, and the oe-LINC00261 group examined by CCK-8 assay. **E** Cell cycle distribution of PANC-1 cells and stem cells overexpressing LINC00261 examined by flow cytometry. **F** Cell sphere formation ability of PANC-1 cells and stem cells overexpressing LINC00261. **G** Invasion of PANC-1 cells and stem cells overexpressing LINC00261 assessed by Transwell assay. **H** Cytotoxicity of PANC-1 cells and stem cells overexpressing LINC00261. **I** Tumor volume in nude mice bearing PANC-1 cells and stem cells overexpressing LINC00261. *p < 0.05 vs. the oe-NC group in panel A or PANC-1 cells in panels B-I. ^#^*p* < 0.05 vs. PANC-1 stem cells. Each experiment was repeated three times and measurement data were expressed as mean ± standard deviation. Comparisons between two groups were analyzed by independent sample *t* test, comparisons of data and different time point were analyzed by repeated measures ANOVA followed by Bonferroni post hoc test, and comparisons among multiple groups were analyzed by one-way ANOVA, followed by Tukey’s post hoc test. CCK-8 cell counting kit-8.

The transfection efficiency of a LINC00261 overexpression plasmid (oe-LINC00261) in PANC-1 stem cells was determined by RT-qPCR. As shown in Fig. 3A, compared to the oe-negative control (NC) group, the expression of LINC00261 increased in the oe-LINC00261 group (*p* < 0.05). We then examined how increased expression of LINC00261 in PANC-1 cells and stem cells affected mRNA and protein expression of stem cell markers (Nanog, Oct4, Sox2, CDl33, and EpCAM), cell proliferation, and cell cycle distribution by adopting RT-qPCR, cell counting kit-8 (CCK-8) assay, and flow cytometry assays, respectively. The results identified an increase in the expression of stem cell markers (Fig. 3B, C), enhanced cell viability (Fig. 3D), and reduced cell proportion in G0/G1 phrase (Fig. 3E) in PANC-1 stem cells relative to PANC-1 cells. All these indicators were observed to have reverse tendency when the LINC00261 was overexpressed in PANC-1 stem cells (Fig. 3B–E).

Moreover, the effect of the overexpression of the LINC00261 on the self-renewal ability, cell invasion, drug resistance, and tumorigenicity of PANC-1 stem cells was assessed by means of cell sphere formation assay (Fig. 3F), Transwell assay (Fig. 3G), cytotoxicity test (Fig. 3H), and tumor xenograft in nude mice (Fig. 3I). These assays showed that PANC-1 stem cells exhibited enhanced cell sphere formation ability, invasive ability, resistance to gemcitabine, and tumorigenic ability when compared to PANC-1 cells, whereas the cell sphere formation ability, invasive ability, resistance to gemcitabine, and the tumor volume were suppressed when the LINC00261 was overexpressed in PANC-1 stem cells. These results indicate that restoration of LINC00261 expression in PANC-1 stem cells could be used to inhibit the proliferation, self-renewal, invasion and tumorigenicity, and increase sensitivity to gemcitabine of PANC-1 stem cells.

### LINC00261 increases ITIH5 expression by recruiting GATA6

**Fig. 4:**
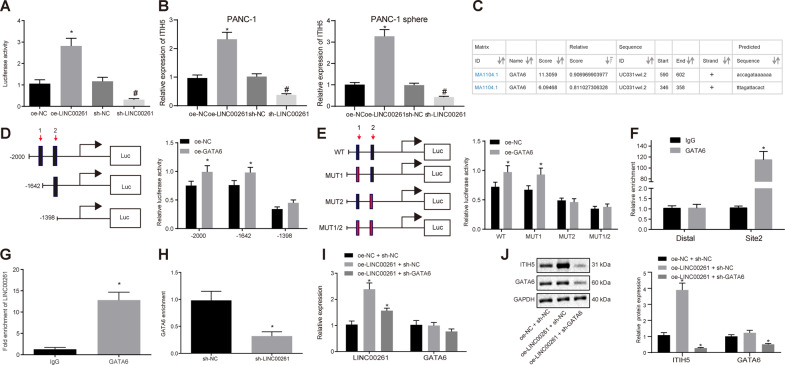
LINC00261 upregulates ITIH5 expression by recruiting GATA6. **A** The effect of LINC00261 on the activity of ITIH5 promoter in the PANC-1 spheres, examined by dual-luciferase reporter gene assay; *p < 0.05 vs. the oe-NC group; ^#^*p* < 0.05 vs. the sh-NC group. **B** Expression of ITIH5 in PANC-1 cells and spheres examined by RT-qPCR, *p < 0.05 vs. the oe-NC group; ^#^*p* < 0.05 vs. the sh-NC group. **C** Two potential binding sites of GATA6 and ITIH5 DNA predicted by UCSC and JASPAR. **D** Cotransfection of truncated ITIH5 recombinant luciferase reporter gene vector and GATA6 overexpression vector into PANC-1 stem cells; *p < 0.05 vs. the oe-NC group. **E** Cotransfection of mutant ITIH5 recombinant luciferase reporter gene vector and GATA6 expression vector into PANC-1 stem cells; *p < 0.05 vs. the oe-NC group. **F** Binding of GATA6 in site 2 of ITIH5 DNA examined by ChIP-qPCR assay; *p < 0.05 vs. the IgG group. **G** The interaction between LINC00261 and GATA6 verified by RIP assay; *p < 0.05 vs. the IgG group. **H** The regulatory effect of LINC00261 on GATA6 enrichment examined by ChIP-qPCR assay; *p < 0.05 vs. sh-NC group. **I** Transfection efficiency examined by RT-qPCR; *p < 0.05 vs. the oe-NC + sh-NC group. **J** The expression of ITIH5 and GATA6 in each group assessed by western blot analysis; *p < 0.05 vs. the oe-NC + sh-NC group. Each experiment was repeated three times and measurement data were expressed as mean ± standard deviation. Comparisons between two groups were analyzed by independent sample *t* test and comparisons among multiple groups were analyzed by one-way ANOVA, followed by Tukey’s post hoc test. RIP RNA-binding protein immunoprecipitation.

The regulatory effect of LINC00261 on ITIH5 promoter was analyzed by a dual-luciferase reporter assay. The results displayed that, compared with the oe-NC group, the activity of ITIH5 promoter was increased in the oe-LINC00261 group (*p* < 0.05, Fig. 4A). Besides, compared with the short hairpin RNA (sh)-NC group, the activity of ITIH5 promoter was decreased in the sh-LINC00261 group (*p* < 0.05). In both PANC-1 cells and PANC-1 spheres, overexpressed LINC00261 led to upregulated ITIH5 expression, while silenced LINC00261 resulted in downregulated ITIH5 expression (*p* < 0.05, Fig. 4B). These results indicate that LINC00261 upregulates ITIH5 expression.

In order to determine how LINC00261 upregulates ITIH5 expression, we hypothesized that GATA6 may play a role. UCSC (http://genome.ucsc.edu/) and JASPAR (http://jaspar.genereg.net/) websites were used to determine the GATA6 binding site in ITIH5 DNA promoter, identifying 2 potential binding sites of GATA6 in the ITIH5 promoter region (Fig. 4C). Dual-luciferase reporter gene assay was conducted to verify the binding site in vitro. Compared with the oe-NC group, the activity of ITIH5 in the oe-GATA6 group was decreased significantly when the binding site 2 of the promoter was truncated or mutated (*p* < 0.05), but the activity of ITIH5 was not affected when the binding site 1 of the promoter was truncated or mutated (*p* > 0.05) (Fig. 4D, E). This indicated that site 2 is the functional site at which GATA6 binding to ITIH5 DNA promoter. The interaction between GATA6 and ITIH5 DNA promoter was subsequently confirmed by chromatin immunoprecipitation (ChIP)-qPCR assay and the results revealed that compared with the immunoglobulin G (IgG) group, the amplified products from site primer were more than that from distal primers in the GATA6 group (*p* < 0.05), while there was no significant change in regard to these two primers in the IgG group (*p* > 0.05) (Fig. 4F). Therefore, it appears that site 2 of ITIH5 DNA promoter (ACCAGATAAAAAA) is a regulatory binding site of GATA6 to ITIH5. RNA-binding protein immunoprecipitation (RIP) assay was subsequently performed to determine whether LINC00261 could bind to GATA6. The results displayed that, compared with the IgG group, the LINC00261 expression coprecipitation with GATA6 was increased (*p* < 0.05) (Fig. 4G), suggesting that GATA6 can specifically bind to LINC00261. In addition, the role of LINC00261 in regulating GATA6 and ITIH5 was determined by ChIP assay and the results showed the enrichment of GATA6 on site 2 of ITIH5 promoter was decreased in the sh-LINC00261 group (*p* < 0.05) (Fig. 4H), suggesting that GATA6 can promote the expression of ITIH5 through binding to its promoter at site 2 in the presence of LINC00261.

To further demonstrate whether LINC00261 regulates the expression of ITIH5 by recruiting the transcription factor GATA6, the PANC-1 stem cells were transfected with oe-LINC00261 with or without transfection with sh-GATA6, and the transfection efficiency was determined by RT-qPCR. The results revealed that compared with the oe-NC + sh-NC group, the transfection was effective in the oe-LINC00261 + sh-NC and oe-LINC00261 + sh-GATA6 groups (all *p* < 0.05) (Fig. 4I). The expression of ITIH5 following cell transfection was then determined by RT-qPCR and western blot analysis and the results showed that, when compared with the oe-NC + sh-NC group, the expression of ITIH5 was increased in the oe-LINC00261 + sh-NC group (*p* < 0.05) but decreased in the oe-LINC00261 + sh-GATA6 group (*p* < 0.05). In contrast, the expression of GATA6 was not changed in the oe-LINC00261 + sh-NC group, but distinctly reduced the oe-LINC00261 + sh-GATA6 group (*p* < 0.05) (Fig. 4J), indicating that LINC00261 upregulated ITIH5 expression by recruiting GATA6.

### ITIH5 inhibits the proliferation, self-renewal, invasion, and tumorigenicity of PC stem cells while also enhancing sensitivity to gemcitabine

**Fig. 5:**
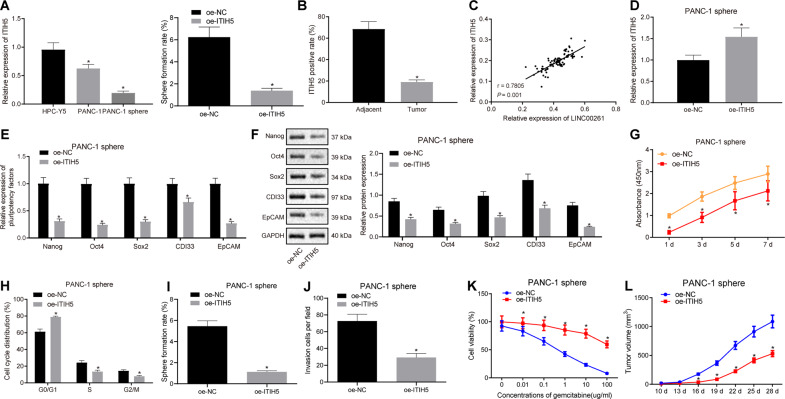
Upregulation of ITIH5 suppresses the proliferation, self-renewal, invasion, and tumorigenicity of PC stem cells while enhancing their sensitivity to gemcitabine treatment. **A** ITIH5 expression in PANC-1 stem cells examined by RT-qPCR, *p < 0.05 vs. the HPC-Y5 cells. **B** ITIH5 expression in PC and adjacent normal tissues, *p < 0.05 vs. the adjacent normal tissues (200×). **C** Correlation analysis between LINC00261 and ITIH5. **D** The transfection efficiency of oe-ITIH5 in PANC-1 stem cells determined by RT-qPCR. **E** The expression of stem cell markers in PANC-1 stem cells overexpressing ITIH5 determined by RT-qPCR. **F** The protein expression of stem cell markers in PANC-1 stem cells studied by western blot analysis. **G** Proliferation of PANC-1 stem cells overexpressing ITIH5 examined by CCK-8 assay. **H** Effect of ITIH5 overexpression on cell cycle distribution in PANC-1 stem cells examined by flow cytometry. **I** Cell sphere formation ability of PANC-1 stem cells. **J** Invasion of PANC-1 stem cells assessed by Transwell assay. **K** Cytotoxicity of PANC-1 cells. **L** Tumor volume in nude mice bearing PANC-1 stem cells overexpressing ITIH5, *n* = 5. *p < 0.05 vs. the oe-NC group (in **D**–**L**). Each experiment was repeated three times and measurement data were expressed as mean ± standard deviation. Comparisons between two groups were analyzed by paired or independent sample *t* test, comparisons of data and different time point were analyzed by repeated measures ANOVA followed by Bonferroni post hoc test, and comparisons among multiple groups were analyzed by one-way ANOVA, followed by Tukey’s post hoc test.

Given that the above results indicate that LINC00261 binds to GATA6 to upregulate the expression of ITIH5, we decide to turn our attention to expanding the role ITIH5 plays in PC. The expression of ITIH5 in PANC-1 cells, PANC-1 stem cells, and HPC-Y5 cells was determined by RT-qPCR, revealing that, when compared with HPC-Y5 control cells, the ITIH5 expression was significantly lower in PANC-1 cells, PANC-1 stem cells (*p* < 0.05), with the lowest levels detected in the PANC-1 stem cells (*p* < 0.05) (Fig. 5A). Next, the expression of ITIH5 in PC tissues was determined by immunohistochemistry, revealing that compared with the adjacent normal tissues, the ITIH5 expression is lower in PC tissues in vivo (*p* < 0.05) (Fig. 5B). Correlation analysis was then carried out to analyze the association between LINC00261 and ITIH5 in PC tissues, results of which showed that the expression of LINC00261 positively correlates with the expression of ITIH5 in PC cells (Fig. 5C).

Next, the biological function of low expression of ITIH5 in PANC-1 stem cells was examined by overexpressing ITIH5 in PANC-1 stem cells. The transfection efficiency of oe-ITIH5 in PANC-1 stem cells was confirmed by RT-qPCR with ITIH5 expression clearly increased in the oe-ITIH5 group compared with that in the oe-NC group (*p* < 0.05) (Fig. 5D). Examination of this model system also showed that the mRNA and protein expression of stem cell markers was decreased (Fig. 5E, F), the cell proliferation was inhibited (Fig. 5G), and the proportion of cells in G0/G1 phrase increased (Fig. 5H) in the PANC-1 stem cells after overexpression of ITIH5. Moreover, cell sphere formation ability (Fig. 5I), the number of cells penetrated the membrane (Fig. 5J), resistance to gemcitabine (Fig. 5K), and the tumor volume (Fig. 5L) were suppressed by ITIH5 overexpression in the PANC-1 stem cells (*p* < 0.05). Therefore, ITIH5 overexpression impeded the proliferation, self-renewal, invasion, and tumorigenicity of PANC-1 stem cells, and enhanced their sensitivity to gemcitabine.

### LINC00261 reduces PC stem cell tumorigenicity by recruiting GATA6

**Fig. 6:**
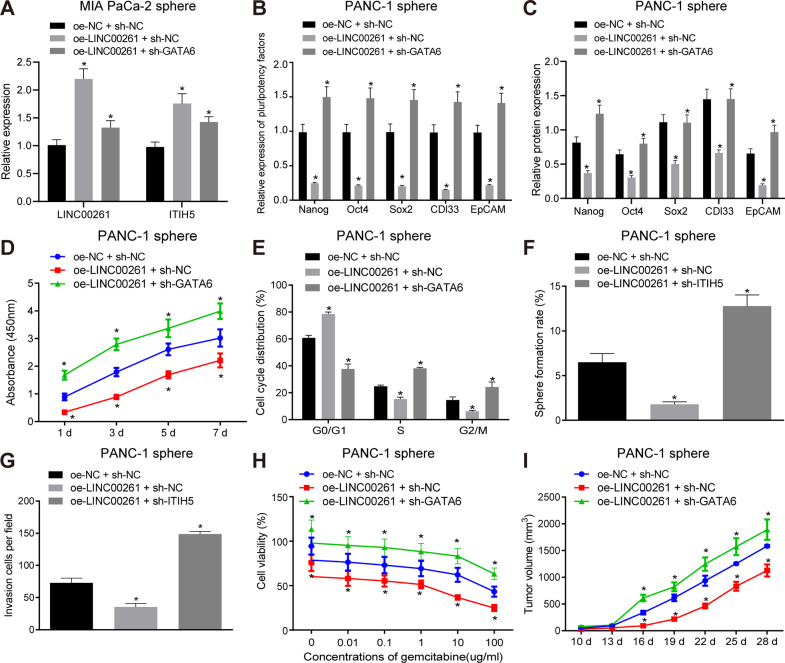
Upregulation of LINC00261 reduces the tumorigenicity of PC stem cells by recruiting GATA6. **A** Expression of LINC00261 and ITIH5 determined by RT-qPCR. **B** mRNA expression of stem cell markers in PANC-1 stem cells determined by RT-qPCR. **C** Protein expression of stem cell markers in PANC-1 stem cells analyzed by western blot analysis. **D** Proliferation of PANC-1 stem cell examined by CCK-8 assay. **E** Cell cycle distribution of PANC-1 stem cells examined by flow cytometry. **F** Cell sphere formation ability of PANC-1 stem cells. **G** Invasion of PANC-1 stem cells assessed by Transwell assay. **H** Cytotoxicity of PANC-1 cells. **I** Tumor volume in nude mice bearing PANC-1 stem cells, *n* = 5. *p < 0.05 vs. the oe-NC + sh-NC group. Each experiment was repeated three times and measurement data were expressed as mean ± standard deviation. Comparisons of data and different time point were analyzed by repeated measures ANOVA followed by Bonferroni post hoc test, and comparisons among multiple groups were analyzed by one-way ANOVA, followed by Tukey’s post hoc test.

The regulatory mechanism by which LINC00261 alters the characteristics of PC stem cells via ITHI5 was investigated using PANC-1 stem cells that have been transfected with oe-LINC00261 with or without sh-GATA6 cotransfection. RT-qPCR analysis confirmed that the overexpression was achieved in the oe-LINC00261 + sh-NC and oe-LINC00261 + sh-GATA6 groups ((*p* > 0.05, Fig. 6A). As depicted in Fig. 6B–E, compared with the oe-NC + sh-NC group, the expression of stem cell markers was decreased, the cell proliferation was inhibited, and the cell in G0/G1 phrase increased in the oe-LINC00261 + sh-NC group (*p* < 0.05), while all these effects of oe-LINC00261 on PANC-1 stem cells were reversed by sh-GATA6 (*p* < 0.05). Moreover, compared with the oe-NC + sh-NC group, the cell sphere formation ability, the invasive ability, the resistance to gemcitabine, and the tumor growth were restrained in the oe-LINC00261 + sh-NC group (*p* < 0.05), while those abilities of PANC-1 stem cells affected by oe-LINC00261 were rescued by sh-GATA6 (*p* < 0.05) (Fig. 6F–I).

Collectively, these findings indicate that LINC00261 represses the tumorigenicity properties of PC stem cells by regulating GATA6.

### LINC00261-mediated recruitment of GATA6 upregulates ITIH5 to promote OC tumorigenicity

**Fig. 7:**
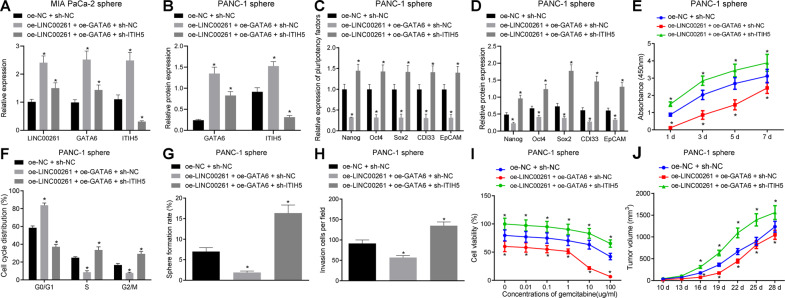
Overexpression of LINC00261 reduces tumorigenicity PC stem cells by increasing ITIH5 via binding to GATA6. **A** Transfection efficiency of ITIH5 silencing in PANC-1 stem cells determined by RT-qPCR. **B** The transfection efficiency of ITIH5 silencing, LINC00261, and GATA6 overexpressing in PANC-1 stem cells measured by western blot analysis. **C** The expression of stem cell markers in PANC-1 stem cells after ITIH5 silencing in the presence of LINC00261 and GATA6 detected by RT-qPCR. **D** The protein expression of stem cell markers in PANC-1 stem cells after ITIH5 silencing in the presence of LINC00261 and GATA6 measured by western blot assay. **E** Proliferation of PANC-1 stem cells after ITIH5 silencing in the presence of LINC00261 and GATA6 examined by CCK-8 assay. **F** Cell cycle distribution of PANC-1 stem cells after ITIH5 silencing in the presence of LINC00261 and GATA6 examined by flow cytometry. **G** Cell sphere formation ability of PANC-1 stem cells. **H** Invasion of PANC-1 stem cells assessed by Transwell assay. **I** Cytotoxicity of PANC-1 cells. **J** Tumor volume in nude mice bearing PANC-1 stem cells, *n* = 5. *p < 0.05 vs. the oe-NC + sh-NC group. Each experiment was repeated three times and measurement data were expressed as mean ± standard deviation. Comparisons of data and different time point were analyzed by repeated measures ANOVA followed by Bonferroni post hoc test, and comparisons among multiple groups were analyzed by one-way ANOVA, followed by Tukey’s post hoc test.

To further explore the mechanism by which LINC00261, GATA6, and ITIH5 regulate the biological properties of PC stem cells, PANC-1 stem cells were transfected with oe-NC + sh-NC, oe-LINC00261 + oe-GATA6 + sh-NC, and oe-LINC00261 + oe-GATA6 + sh-ITIH5. The transfection efficiency was confirmed by RT-qPCR and western blot analysis (Fig. 7A, B). As shown in Fig. 7C–E, compared with the oe-NC + sh-NC group, the expression of stem cell markers was decreased, the cell proliferation rate was inhibited, and the number of cells in G0/G1 phrase was increased in the oe-LINC00261 + oe-GATA6 + sh-NC group (*p* < 0.05). In contrast, increased expression of stem cell markers, enhanced cell proliferation, and reduced cells in G0/G1 phrase were found in the oe-LINC00261 + oe-GATA6 + sh-ITIH5 group (*p* < 0.05). Moreover, compared with the oe-NC + sh-NC group, the cell sphere formation ability, the cell penetrated the membrane, and the tumor volume were decreased, but the sensitivity to gemcitabine was enhanced in the oe-LINC00261 + oe-GATA6 + sh-NC group (*p* < 0.05), whereas these were all reversed in the oe-LINC00261 + oe-GATA6 + sh-ITIH5 group (*p* < 0.05) (Fig. 7F–I).

These results clearly show that LINC00261 reduces the tumorigenic features of PC stem cells by upregulating ITIH5 expression via recruiting GATA6.

## Discussion

PC, with its poor prognosis, is a significant health burden globally [32]. Recent studies have demonstrated that lncRNAs play a role in the progression in various cancers including PC [33]. This study aimed to investigate the role of LINC00261 in PC progression. Based on the experimental results, we found that upregulation of LINC00261 has tumor suppressive functions in PC stem cells through a regulatory mechanism that LINC00261 increased expression ITIH5 by recruiting GATA6.

In this study, we found that LINC00261 expression is reduced in both PC cells and PC stem cells. Similarly, the lncRNA activated by transforming growth factor beta, downregulated in PC tissues and cells, has been shown to correlate with a poor survival prognosis [34]. Also, downregulation of LINC00261 is also correlated with the poor prognosis and inhibits cancer metastasis in gastric cancer [35]. Moreover, reduced expression of LINC00261 in hepatocellular carcinoma (HCC) tissues is associated with a reduced tumor-free survival time [36]. Consistent with our findings, low LINC00671 expression has been shown in PC tissues and serums [31]. Importantly, upregulation of LINC00261 could suppress cell proliferation and metastasis in several human cancers such as choriocarcinoma, gastric cancer, and HCC [29, 37, 38]. Thus, we speculated that LINC00261 overexpression might also be beneficial to prevent PC progression. Indeed, gain-of-function experiments demonstrated that expression of LINC00261 exerts an inhibitory role in the progression of PC by suppressing the proliferation, invasion, and metastasis of PC stem cells.

The results of this study further verified that LINC00261 suppresses the cell self-renewal ability, while it enhanced the sensitivity to gemcitabine in PC stem cells. It has been revealed that lncRNAs have been implicated in the self-renewal of liver CSCs [39, 40].

The transcription factors Nanog, Oct4, and Sox2 have been reported to be essential in promoting embryonic stem cell self-renewal and could be regulated by lncRNAs in an epigenetic network [41]. Sox2 has revealed to be involved in cell metastasis and apoptosis, and its abnormal increase in PC resulted in cell growth and stemness [42, 43]. Interestingly, the results of this study further confirmed that the expressions of Nanog, Oct4, Sox2, and EpCAM were reduced in LINC00261-expressing PC stem cells, showing that the carcinogenic potency was suppressed by overexpressed LINC00261. The transcription factors Nanog, Oct4, and Sox2 were reported to be essential in promoting embryonic stem cell self-renewal and could be regulated by lncRNAs in an epigenetic network [41]. Sox2 has revealed to be involved in cell metastasis and apoptosis, and its abnormal increase in PC resulted in cell growth and stemness [42, 43]. LncRNAs have also been demonstrated to mediate drug resistance in PC [44]. For instance, lncRNA taurine-upregulated gene 1 is proposed to enhance gemcitabine resistant in pancreatic ductal adenocarcinoma [45]. Moreover, induction of lncRNA HOTAIR confers resistance and malignancy of PC stem cells during the course of gemcitabine treatment [46]. Similar with our data, LINC00261 sensitizes human esophageal cancer cells to 5-fluorouracil [47] and colon cancer cells to cisplatin [48]. These findings reveal a promising potential therapeutic target in the treatment of PC, namely the targeting of LINC00261 during gemcitabine treatment for PC. However, this application warrants further exploration due to insufficient current clinical data to support this.

Finally, this study also demonstrated that LINC00261 increases the ITIH5 expression via binding to GATA6, which might be the potential regulatory network in PC progression. Transcription factors, miRNAs and lncRNAs have been shown to interreact in transcriptional regulatory circuitry for the maintenance of embryonic stem cell stemness [49]. A systematic analysis reveals novel lncRNA/transcription factor regulatory pairs such as LINC00261/FOXA2 and GATA6-AS1/GATA6 involving in tumorigenesis [50]. Poorly expressed ITIH5 was also observed in pancreatic ductal adenocarcinoma, while its restoration could also display an inhibitory function in metastasis of PC cells [51]. A previous study has reported that a lncRNA MIR31HG-induced ITIH5 activation could inhibit migration and proliferation in Hirschsprung’s disease [52]. Moreover, a study has revealed that GATA6 is essential in inhibiting EMT and tumor growth and is considered as a crucial biomarker in the progression of PC [53]. The loss- and gain-of-function studies led to the conclusion that ectpically overexpressed LINC00261 and ITIH5 inhibited the cell proliferation, invasion and tumorigenicity in PC stem cells, but enhanced the sensitivity to gemcitabine. We also found that the loss of ITIH5 reversed the inhibition of LINC00261 and GATA6 on the tumorigenicity in vitro and in vivo. Collectively, these findings indicate that upregulation of LINC00261 attenuates the properties of PC stem cells by upregulating ITIH5 through recruiting GATA6.

**Fig. 8:**
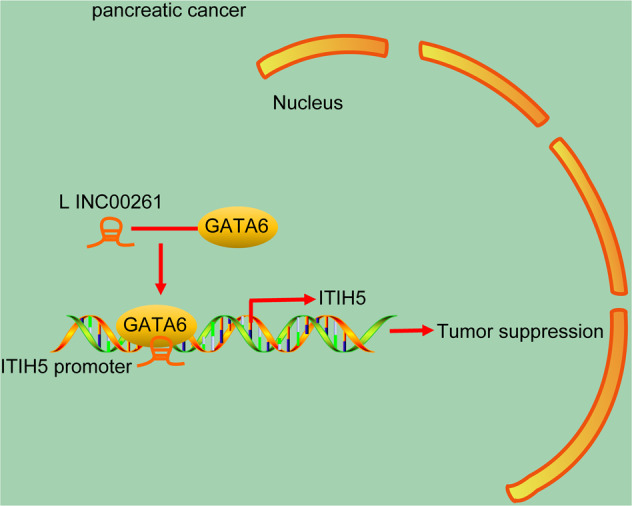
The molecular mechanism by which LINC00261 acts in PC. In the presence of LINC00261, GATA6 is recruited to the ITIH5 promoter region, promoting ITIH5 expression and thereby attenuating biological characteristics such as cell proliferation, self-renewal, and invasion and promotes sensitivity to gemcitabine of PC stem cells and ultimately reducing their tumorigenicity.

In summary, the findings from this study demonstrate that LINC00261 inhibits the malignancy of PC stem cells and antagonizes the chemoresistance of PC stem cells. As summarized in Fig. 8, we have identified a molecular mechanism in which LINC00261 increases ITIH5 expression by recruiting GATA6 on the ITIH5 promoter region, thereby attenuating tumorigenicity while enhancing sensitivity to gemcitabine of PC stem cells. This study provides novel targets for the treatment of this devastating disease. However, the development of lncRNA-targeted therapies is challenged by few obstacles, such as the successful delivery of single- or multitarget agents to the target tissues. Therefore, following-up experiments are required to further confirm the functions of LINC00261.

## Materials and methods

### Ethics statement

All the clinical experiments in this study were in strict accordance with the Helsinki declaration and followed the principles of the ethics committee in The Fourth Affiliated Hospital of China Medical University. The written consent was obtained from all participants or their guardians. The protocol of animal experiments was approved by the Institutional Animal Care and Use Committee of The Fourth Affiliated Hospital of China Medical University.

### Microarray gene expression profiling

PC-related expression datasets were downloaded from the Gene Expression Omnibus database (https://www.ncbi.nlm.nih.gov/geo/). R language affy package was applied for the standardization pretreatment of the expression datasets [54]. Differentially expressed lncRNAs and genes in PC tissues and normal tissues were filtered using limma package with |log2FC| > 1.5 and adj.P.Val < 0.05 (the corrected *p* value was expressed as adj.P.Val) [55]. The differentially expressed lncRNAs and genes were plotted in heatmap.

### Tissue collection and cell line selection

PC and adjacent normal tissues were collected from 88 PC patients who had undergone tumor resection at The Fourth Affiliated Hospital of China Medical University during March 2015 and May 2018.

Five PC cell lines including PANC-1, AsPC-1, HS 766T, SW1990, and MIA PaCa-2 and normal pancreatic epithelial cell line HPC-Y5 were purchased from the National Infrastructure of Cell Line Resource (http://www.cellresource.cn/). The cells were incubated in complete Dulbecco’s modified Eagle’s medium (DMEM; Gibco, Carlsbad, CA, USA) supplemented with 10% fetal bovine serum (FBS), 100 µg/mL streptomycin, and 100 U/mL penicillin at 37 °C with 5% CO_2_ under 95% saturated humidity. Cells were split when cell confluence reached 80%. The expression of LINC00261 in each cell line was determined by RT-qPCR and the cell line with the lowest expression of LINC00261 was employed for further experiment.

### Cell transfection

The oe-NC, LINC00261, ITH5, or GATA6 overexpression plasmid (oe-LINC00261, oe-ITH5, and oe-GATA6), scramble shRNA (sh-NC), and shRNAs targeting LINC0261, GATA6, or ITH5 (sh-LINC0261, sh-GATA6, and sh-ITH5) were purchased from Dharmacon (Lafayette, CO, USA). The cells were seeded on a six-well plate and when cell density reached 70–80% on the second day, the cell transfection was carried out using Lipofectamine 2000 (Invitrogen, Carlsbad, CA, USA). For transfection, 4 µg target plasmid and 10 µL Lipofectamine 2000 were separately diluted with 250 µL Opti-MEM (Gibco, Carlsbad, CA, USA) and allowed to stand for 5 min at room temperature. Then, the two dilutions were mixed and allowed to stand at room temperature for 20 min before adding into wells. After 8 h of transfection, the culture medium was replaced by fresh culture medium and the cells were treated with 400 µg/mL G418 after 48 h transfection. The culture medium was replaced every 3 day and the dosage of G418 was reduced for maintenance until the stable transfected cell lines were obtained.

### Isolation of PC stem cells

A standard cell culture medium (Gibco, Carlsbad, CA, USA) consisted of serum-free DMEM with addition of hepatocyte growth factor (10 ng/mL), basic fibroblast growth factor (bFGF) (10 ng/mL), and Noggin (10 ng/mL) + LIF (1000 U/mL). PANC-1 cells or MIA PaCa-2 cells were seeded in 96-well plate (ultralow plates, Becton, Dickinson and Company, NJ, USA), with 2 mL DMEM containing 10% FBS at 37 °C with 5% CO_2_. Half culture medium was replaced by fresh culture medium every 2 day and the subculture was carried out once the cell sphere diameter was larger than 50 μm. The adhered cells were abandoned during the subculture and the suspended cells were collected by centrifugation at 300 × *g* for 3 min. After removing the supernatant, cells were treated with 0.25% trypsin and 0.02% ethylene glycol-bis (β-aminoethyl ether)-N, N-tetraacetic acid. The cells were subsequently gently triturated with fresh medium into single-cell suspension. After being centrifuged at 300 × *g* for 3 min, the supernatant was discarded, and the collected cells were cultured with fresh culture medium. After counting, the cells were seeded to a new six-well plate at a density of 1 × 10^4^ cell/well and the subculture was kept for 2 weeks continuously.

### RT-qPCR

Total RNA was extracted using TRIzol reagent (15596026, Invitrogen, Car, USA) and then reversely transcribed into cDNA in accordance with a reverse transcription kit as per the manufacturers protocol (RR047A, Takara, Japan). When cDNAs were ready, RT-qPCR was conducted using SYBR Premix EX Taq kit (RR420A, Takara, Japan) on a real-time quantitative PCR instrument (ABI7500, Foster City, CA, USA). The reaction mixture consisted of 9 µL SYBR Mix, 0.5 µL forward primer, 0.5 µL reversed primer, 2 µL cDNA, and 8 µL RNase Free ddH_2_O. Reaction condition: pre denaturation at 95 °C for 10 min, 40 cycles of denaturation at 95 °C for 15 s, and annealing at 60 °C for 1 min. Three replicates were tested for each sample. All primers (Table S1) were synthesized by Sangon biotechnology Co., Ltd (Shanghai, China). Glyceraldehyde-3-phosphate dehydrogenase (GAPDH) was taken as internal reference control. The relative expression of target genes was calculated using the 2-ΔΔCt method: ΔΔCt = Ct (target gene in the experimental group – house-keeping gene in the experimental group) – Ct (target gene in the control group – house-keeping gene in the control group).

### Western blot analysis

Total protein in tissues or cells was extracted by radio-immunoprecipitation assay (RIPA) lysis buffer supplemented with phenylmethanesulfonyl fluoride. Cells were incubated in RIPA buffer for 4 °C for 30 min, and then centrifuged at 8000 × *g* for 10 min after which the supernatant was collected. A total of 50 µg proteins were mixed with 2 × sodium dodecyl sulfate (SDS) loading buffer, boiled at 100 °C for 5 min, separated by SDS-polyacrylamide gel electrophoresis (PAGE), and then transferred to a polyvinylidene fluoride (PVDF) membrane using wet-transferring method. The PVDF membrane was then sealed with 5% skim milk at room temperature for 1 h, followed by incubation with diluted primary antibody to ITH5 (ab107846, 1:100) overnight at 4 °C with GAPDH (ab9485, 1:2500) as internal reference. The membrane was washed with Tris-buffered saline Tween-20 (TBST) three times (10 min for each) on the next day and subsequently incubated with secondary antibody horseradish peroxidase-labeled goat anti-rabbit IgG (ab97051, 1:2000) for 1 h. After washed with TBST, the membrane was developed using enhanced chemiluminescence reagent (BB-3501, Amersham, Little Chalfont, UK). The membrane was then exposed to a gel imager and the Bio-Rad image analysis system (Bio-Rad, Richmond, CA, USA) was employed for photography, while Quantity One (v4.6.2) (Bio-Rad) was used for image analysis. The gray value of protein band to GAPDH was considered as the relative protein expression. All the antibodies were purchased from Abcam (Cambridge, UK).

### FISH

The subcellular localization of LINC00261 was determined by FISH analysis as explained previously [56], following the instruction of Ribo^TM^ lncRNA FISH Probe Mix (Red) Kit (C10920, RiboBio, Shenzhen, China). Briefly, the cell slide was seeded in a 24-well plate at a density of 6 × 10^4^ cells/well until the cell confluence reached 60–70%. Subsequently, cells were fixed by 1 mL 4% paraformaldehyde at room temperature for 10 min and permeabilized with 1 mL precooled phosphate buffer saline (PBS) containing 0.5% Triton X-100 at 4 °C for 5 min. After PBS washing, cells in each well were sealed with 200 µL prehybridization solution at 37 °C for 30 min, and then hybridized with nucleotide probe against LINC00261 (GeneCreate, Wuhan, China) at 37 °C overnight without light. Cells were rinsed in Lotion I (4 × SSC, 0.1% Tween-20), Lotion II (2 × SCC), Lotion III (1 × SCC), respectively, and then washed with 1 × PBS three times (5 min for each). Finally, cells were stained in 4′,6-diamidino-2-phenylindole solution (1: 800) for 10 min, washed, and sealed with nail enamel. Five visual fields selected at random, cells were observed and imagined under a fluorescence microscope (Olympus, Japan).

### Immunohistochemistry

PC tissues were fixed with 4% paraformaldehyde, dewaxed paraffin embedded, and sliced into sections. Immunohistochemical assessment of ITIH5 expression was conducted as previously described [57]. Briefly, tissue sections were incubated with 50 µL ITIH5 primary antibody (ab107846, 1:50, Abcam, Shanghai, China) at room temperature for 1 h and then washed with PBS. Thereafter, 50 µL secondary antibody goat anti-rabbit IgG (ab6721, 1:1000, Shanghai, China) was added for incubation at room temperature for 1 h, followed by PBS washing. Next, the sections were stained with streptavidin peroxidase at 37 °C for 30 min. After PBS washing, the sections were stained in diaminobenzidine for 5–10 min, and then counterstained by hematoxylin for 2 min. The sections were differentiated by hydrochloric ethanol, washed in water for 10 min. The staining results were observed under a microscope.

### RIP assay

The binding of LINC00261 to GATA6 was determined using a RIP kit (Millipore, Billerica, MA, USA). Cells were lysed by lysis buffer with equal volume for 5 min on ice-bath and then centrifuged at 14,000 × *g* at 4 °C for 10 min to collect the supernatant. Meanwhile, 1 µg antibody was coupled to 50 µL magnetic bead (washed and resuspended in 100 µL RIP Wash buffer). The magnetic bead-antibody compound was resuspended in 900 µL RIP wash buffer, and subsequently 100 µL cell extracts were coprecipitated with magnetic bead-antibody compound at 4 °C overnight. On the next day, the magnetic bead-protein compound was collected, and then digested by protease K for RNA extraction for further experiment. The antibody used for RIP assay was GATA6 (ab22600, 1:1000, Abcam, Shanghai, China) and IgG (ab172730, 1:100, Abcam, Shanghai, China) was used as NC.

### Dual-luciferase reporter gene assay

The oe-NC, oe-LINC00261, sh-NC, and sh-LINC00261 were cotransfected with ITH5-2Kb into PANC-1 or MIA PaCa-2 stem cells to examine the effect of LINC00261 on the activity of ITIH5 promoter. The cells were collected after 48 h of transfection. Luciferase reporter assay kit (K801-200, BioVision, Milpitas, CA, USA) was employed for luciferase activity detection using a dual-luciferase reporter system (Promega, Madison, WI, USA). Renilla luciferase was taken as internal reference and the activation of the target reporter gene was determined by the ratio of the firefly luciferase activity RLU to the renilla luciferase activity RLU.

The UCSC (http://genome.ucsc.edu/) and JASPAR (http://jaspar.genereg.net/) websites were used to predict the binding sites that GATA6 may bind to ITIH5 promoter. Based on these predictions, recombinant luciferase reporter gene vector with truncated or mutant binding sites was constructed and then cotransfected with GATA6 overexpressing vector into PANC-1 stem cells.

### ChIP assay

The PANC-1 stem cells were fixed with formaldehyde for 10 min, and cross-linked chromatin was sonicated into fragments. The fragments-contained mixture was centrifuged at 12,000 × *g* at 4 °C for 10 min to collect the supernatant, which were immunoprecipitated with GATA6 antibody (Upstate Biotech, Lake Placid, NY, USA) or NC rabbit IgG (ab109489, 1:300, Abcam) at 4 °C for overnight. The next day, DNA–protein complex was precipitated using protein agarose/sepharose and then subjected to centrifugation at 12,000 × *g* for 5 min to remove the supernatant. The nonspecific complex was eluted, and the cross-linking was reversed at 65 °C overnight. The next day, the extraction and purification of DNA fragments was performed on the mixture by phenol or chloroform. The primers containing GATA6 binding site 2 on ITIH5 DNA promoter were designed (Forward: 5′-CTGTCACTCACCTCACCTATTTAG-3′, Reverse: 5′-TATCTTGGTAGCGTGTAGTATTTC-3′) to obtain an amplified product in a length of 279 bp (including sequences containing the GATA6 binding site 2 on ITIH5 DNA promoter, 590–602 bp), which was 1233 bp away from the transcriptional start site (TSS). Meanwhile, a primer that could amplified the sequence far from the ITIH5 DNA promoter region was also designed as NC for primers of site 2 (Forward: 5′-TGAAGCAGAGGTTGAAGGGAG-3′, Reverse: 5′-TTTGAAAGCCAAGTGGAGAAG-3′). The amplified product was in a length of 385 and 4115 bp away from the TSS. The recovered and purified DNA fragments were taken as amplification templates, and site 2 primer and distal primer were added respectively for RT-qPCR to examine whether site 2 in ITIH5 DNA was the binding site of GATA6.

Purified DNA fragments were obtained after silencing LINC00261, and ChIP was performed as aforementioned. In this case, the sh-NC group was taken as control and the primers of site 2 were employed to detect the enrichment of GATA6 in the site 2 on the ITIH5 promoter.

### CCK-8 assay

Stable PANC-1 cells in the logarithmic growth phase were collected and placed on a 96-well plate with 3000 cells/well and 12 wells/treatment. Subsequently, cells were seeded with 100 µL serum-free DMEM (10 ng/mL bFGF + 20 ng/mL EGF + 5 µg/mL insulin + 100 unit/mL penicillin + 100 µg/mL streptomycin), using only medium as a blank control group for zero adjustment. Cells in each well were treated with 10 µL CCK-8 on the 1st, 3nd, 5th, and 7th day and then incubated for 2 h, respectively. The optical density (OD) value at 450 nm was determined using a microplate reader (Bio-Tek, BD, Norcross, GA, USA) and that in three parallel wells was detected to observe the cell growth.

### Flow cytometry

Cells were made into single-cell suspension and then transferred into a 15-mL centrifugation tube for centrifugation at 4 °C at 300 × *g* for 5 min. After the supernatant was discarded, cells were washed with 3 mL precooled PBS twice and the supernatant was discarded after centrifuging at 4 °C at 300 × *g* for 5 min again. The antibodies (BD Biosciences, San Jose, CA, USA) against CD133 (566596) and EpCAM (563477) were added to cells and incubated at room temperature for 15 min, washed by PBS, and resuspended in 100 µL PBS. Next, cells were fixed by 75% precooled ethanol, triturated gently, and then stored in a refrigerator at −20 °C overnight. On the next day, cells were collected by centrifugation at 4 °C at 300 × *g* for another 5 min with the supernatant discarded. Cells were then washed with 3 mL precooled PBS, and the centrifuged again to discard the supernatant. Finally, cells were incubated with 400 µL PBS, 50 µL RNA enzyme (1 mg/mL), and 10 µL propidium iodide, respectively, at room temperature without light for 30 min before detection.

### Cell sphere formation assay

PANC-1 cells were detached, centrifuged, and washed with PBS once and then the cells were resuspended in FBS-free DMEM (10 ng/mL bFGF + 20 ng/mL EGF + 5 µg/mL insulin + B27 + 100 unit/mL penicillin + 100 µg/mL streptomycin) and the cell density was adjusted to 1 × 10^5^ cells/mL. Next, 1 mL single-cell suspension and the above FBS-free DMEM were added into 25 cm^2^ ultralow adsorption culture bottle (the total volume reached 8 mL), and then the cells were incubated in an incubator with 5% CO_2_ at 37 °C. Finally, five visual fields were randomly chosen to be visualized under an inverted microscope (400×) after 1 week of incubation. The number of cell spheres was counted in each visual field with a volume of ≥40 µm being considered as successful formation of cell sphere. From these counts, the cell sphere formation rate in each group was calculated.

### Transwell assay

Extracellular matrix (ECM) gel was placed at 4 °C overnight, and then diluted into a density of 1 mg/mL with serum-free culture medium (1:9) on the next day. All the pipettes and chambers were precooled on ice for half an hour before experiment. The polycarbonate membrane of apical Transwell chamber (24-well) was added with 40 µL ECM gel and placed at 37 °C with 5% CO_2_ for 5 h for gel formation. The gel was hydrated with 70 µL medium at 37 °C for 0.5 h. PANC-1 or MIA PaCa-2 stem cells were then starved for 24 h in the serum-free medium. Cells were then detached, centrifuged, precipitated, and resuspended in fresh serum-free DMEM to reach the cell density of 2.5 × 10^5^ cells/mL. Next, 0.2 mL cell suspension was added into the apical chamber with hydrated basement membrane and 700 µL DMEM medium containing 10% FBS was added into basolateral chamber. The chambers were incubated with 5% CO_2_ at 37 °C for 24 h and then the cells attached on the chamber and basement membrane were removed using wet cotton bud. After fixing with methanol for 30 min, cells were stained with 0.1% crystal violet before observation. Five visual fields were randomly selected under an inverted microscope, and the number of cells penetrated the membrane was counted.

### Cytotoxicity test

Stable PANC-1 cells in logarithmic growth phase were separated and seeded in a 96-well plate (3000 cells/well; 12 wells per treatment). Serum-free DMEM (10 ng/mL bFGF + 20 ng/mL EGF + 5 µg/mL insulin + B27 + 100 unit/mL penicillin + 100 µg/mL streptomycin) was added into each well, and the well with only medium was used as a blank control. Cells were incubated for 24 h and then subjected to treatment with gemcitabine at different concentrations (0, 0.01, 0.1, 1, 10, 100 µg/mL). After 72 h, 10 µL CCK-8 was added to each well for 2-h incubation. The OD value at 450 nm was determined using a microplate reader (Bio-Tek) and that in three parallel wells was counted and the cell survival rate was calculated.

### Tumor xenograft in nude mice

BALA/c nude mice (aged 4 weeks, weighing 18–23 g) purchased from Hunan SJA Laboratory Animal Co., Ltd. (Changsha, China) were raised in specific pathogen free condition. All nude mice were randomly divided into ten groups with five mice in each group: PANC-1, PANC-1 sphere-NC, PANC-1 sphere-NC + oe-LINC00261, PANC-1 sphere-NC + oe-NC, PANC-1 sphere-NC + oe-ITIH5, PANC-1 sphere-NC + oe-NC + sh-NC, PANC-1 sphere-NC + oe-LINC00261 + sh-NC, PANC-1 sphere-NC + oe-LINC00261 + sh-ITIH5, PANC-1 sphere-NC + oe-LINC00261 + oe-GATA6 + sh-NC, and PANC-1 sphere-NC + oe-LINC00261 + oe-GATA6 + sh-ITIH5, respectively.

According to the treatment conditions outlined above, 100 µL suspension of nontransfected PANC-1 cells or stably transfected PANC-1 spheres (4 × 10^4^) were subcutaneously injected into each mouse and the volume of xenograft tumor was recorded at the 10th day (*V* = [*A* × *B*^2^]/2, [*A*, long diameter; *B*, short diameter; unit: mm^3^]). The tumor volume was measured every 3 days. Mice were then euthanized by carbon dioxide euthanasia after 28 days. The curve of average volume at each time point was plotted.

### Statistical analysis

Statistical analyses were conducted using SPSS 21.0 software (IBM Corp, Armonk, NY, USA). The measurement data were expressed as mean ± standard deviation throughout. Normal distribution and homogeneity of variance were tested. Data of PC tissues and adjacent normal tissues obeying normal distribution and homogeneity of variance were analyzed by paired *t* test, comparisons between two groups were performed using independent sample *t* test and comparisons among multiple groups were analyzed by one-way analysis of variance (ANOVA) or repeated measures ANOVA. Pairwise comparison was tested by post hoc test. Data with skew distribution and heterogeneity of variance were examined with rank sum test. Pearson’s correlation coefficient was used for correlation analysis. *p* < 0.05 was considered statistically significant.

## Supplementary information

Supplemental material

## Data Availability

The datasets generated/analyzed during the current study are available.
