# Supplementary material for: Long noncoding RNA LINC00261 upregulates ITIH5 to impair tumorigenic ability of pancreatic cancer stem cells
Source: Cell Death Discov. 2021 Aug 26;7:220. doi: 10.1038/s41420-021-00575-0 (PMC8390744; doi:10.1038/s41420-021-00575-0)
Supplement: Supplementary file 1 — Supplemental material [file 41420_2021_575_MOESM1_ESM.doc]

T**able S1** Primer sequences for RT-qPCR

| Gene | Primer sequence |
| --- | --- |
| LINC00261 | F: 5'-CCTGAACCTGAGGGAAACTAA-3' |
|  | R: 5'-GCAGAAAGGCACTTGAAAGC-3' |
| ITIH5 | F: 5'-CTTGGATGGTGGGGACAGAC-3' |
|  | R: 5'-TATGGAGCCCTGGATGGTGA-3' |
| GATA6 | F: 5'-TCTCCATGTGCATTGGGGAC-3' |
|  | R: 5'-AAGGAAATCGCCCTGTTCGT-3' |
| GAPDH | F: 5'-TCAGCAATGCCTCCTGCAC-3' |
|  | R: 5'-TCTGGGTGGCAGTGATGGC-3' |
| Nanog | F: 5'-TTGTGGGCCTGAAGAAAACTATCC-3' |
|  | R: 5'-CTGCGTCACACCATTGCTATTCTT-3' |
| Oct4 | F: 5'-GACAACAATGAGAACCTTCAGGAGA-3' |
|  | R: 5'-CTGGCGCCGGTTACAGAACCA-3' |
| Sox2 | F: 5'-ATGCACAACTCGGAGATCAGC-3' |
|  | R: 5'-CCTTCTTCATGAGCGTCTTGG-3' |
| CD133 | F: 5'-ACAGATGCTCCTAAGGCTTG-3' |
|  | R: 5'-CTCCCCGACAGTGCGATG-3' |
| EpCAM | F: 5'-GCTGGCCGTAAACTGCTTTG-3' |
|  | R: 5'-ACATTTGGCAGCCAGCTTTG-3' |

**Notes:** RT-qPCR: reverse transcription quantitative polymerase chain reaction; F, forward; R, reverse; LINC00261, long intergenic non-protein coding RNA 261; ITIH5, inter-alpha-trypsin inhibitor heavy chain 5; GATA6, GATA binding protein 6; GAPDH, glyceraldehyde-3-phosphate dehydrogenase; Oct4, organic cation/carnitine transporter4; Sox2, SRY (sex determining region Y)-box 2; EpCAM, epithelial cell adhesion molecule.
